# Cost-effectiveness of Humanitarian Pediatric Cardiac Surgery Programs in Low- and Middle-Income Countries

**DOI:** 10.1001/jamanetworkopen.2018.4707

**Published:** 2018-11-16

**Authors:** Marcelo Cardarelli, Sumeet Vaikunth, Katie Mills, Thomas DiSessa, Frank Molloy, Elizabeth Sauter, Karen Bowtell, Roslyn Rivera, Andrew Y. Shin, William Novick

**Affiliations:** 1 Inova Children’s Hospital, Fairfax, Falls Church, Virginia; 2 Department of Pediatrics, Stanford University, Stanford, California; 3 Johns Hopkins All Children’s Hospital, St Petersburg, Florida; 4 Department of Pediatrics, University of Kentucky, Lexington; 5 William Novick Global Cardiac Alliance, Memphis, Tennessee; 6 Department of Pediatrics, Stanford University, Palo Alto, California; 7 Department of Surgery, University of Tennessee Health Science Center–Global Surgery Institute, Memphis

## Abstract

**Importance:**

Endorsement of global humanitarian interventions is based on either proven cost-effectiveness or perceived public health benefits. The cost-effectiveness and long-term benefits of global humanitarian pediatric cardiac surgery are unknown, and funding for this intervention is insufficient.

**Objectives:**

To determine the cost-effectiveness of the intervention (multiple 2-week-long humanitarian pediatric cardiac surgery program assistance trips to various low- and middle-income countries [LMICs]) and to produce a measure of the long-lasting effects of global humanitarian programs.

**Design, Setting, and Participants:**

International, multicenter cost-effectiveness analysis of a cohort of children (aged <16 years) undergoing surgical treatment of congenital heart disease during 2015 in LMICs, including China, Macedonia, Honduras, Iran, Iraq, Libya, Nigeria, Pakistan, Russia, and Ukraine. The study also assessed estimated improvement in the United Nations Human Development Indicators (life expectancy, years of schooling, and gross national income) for each individual survivor, as a proxy for long-term benefits of the intervention.

**Main Outcomes and Measures:**

The primary outcome was cost-effectiveness of the intervention. The secondary outcomes were potential gains in life expectancy, years of schooling, and gross national income per capita for each survivor.

**Results:**

During 2015, 446 patients (192 [43%] female; mean [SD] age, 3.7 [5.4] years) were served in 10 LMICs at an overall cost of $3 210 873. Of them, 424 were children. The cost-effectiveness of the intervention was $171 per disability-adjusted life-year averted. Each survivor in the cohort (390 of 424) potentially gained 39.9 disability-adjusted life-years averted, 3.5 years of schooling, and $159 533 in gross national income per capita during his or her extended lifetime at purchasing power parity and 3% discounting.

**Conclusions and Relevance:**

Humanitarian pediatric cardiac surgery in LMICs is highly cost-effective. It also leaves behind a lasting humanitarian footprint by potentially improving individual development indices.

## Key Points

**Question:**

Is humanitarian global pediatric cardiac surgery in low- and middle-income countries cost-effective?

**Findings:**

This economic evaluation found that for a cohort of 424 children who underwent operations in 10 low- and middle-income countries in 2015, the cost-effectiveness of the intervention was $171 per disability-adjusted life-year averted.

**Meaning:**

Humanitarian pediatric cardiac surgery in low- and middle-income countries is highly cost-effective.

## Teaser

This economic evaluation examines the cost-effectiveness of humanitarian pediatric cardiac surgery interventions in low- and middle-income countries (LMICs).

## Introduction

Of the nearly $38 billion donated in 2016 by Western governments, international public health organizations, and philanthropic corporations, most was channeled toward global health interventions that were deemed to be highly cost-effective or good for local public health goals.^1^ Most supported global health interventions involve infectious disease prevention or treatment, improvements in maternal and child care, or other social public health projects (eg, sanitation and clean water).^1,2^ In contrast, “...the development and delivery of surgical and anesthesia care in LMICs [low- and middle-income countries] has been nearly absent from the global health discourse.”^3^ Surgical treatment of congenital heart disease (CHD), a large and poorly met surgical need in those countries, is absent from the global conversation as well.

Accordingly, the primary goal of this study was to investigate the cost-effectiveness in US dollars per unit of health of a humanitarian intervention consisting of global pediatric cardiac surgery program building. The secondary goal was to produce an enhanced quantifier of the impact of global health care humanitarian efforts by considering what we refer to as the *humanitarian footprint*. We defined this measure of value as the potential improvements in the individualized components of the United Nations Human Development Index (HDI)^4,5^: life expectancy (LE), median years of schooling, and gross national income per capita for each survivor of the cohort of children treated during the year of the study.

## Methods

### Design

An institutional review board waiver was obtained by W.N. through his appointment at the University of Tennessee Health Science Center, Global Surgery Institute. Patients gave oral informed consent to the local surgeon prior to surgery. Every effort was made to report the findings according to the Consolidated Health Economic Evaluation Reporting Standards (CHEERS) reporting guideline for economic evaluations.^6^ Baseline data are expressed as means and standard deviations.

### Patient and Country Data

Anonymized demographics and clinical data sets for every patient who underwent an operation by the William Novick Global Cardiac Alliance during calendar year 2015 in an LMIC were downloaded from the patient database kept by the charity. Patients were classified according to their diagnosis and surgical approach into 1 of the 6 component categories of the Risk Adjusted Classification for Congenital Heart Surgery.^7^

Every patient operated on was included in the calculations of the costs of surgery, regardless of age, diagnosis, surgery, and outcome. Patients older than 16 years and those having an unspecified noncardiac operation (Risk Adjusted Classification for Congenital Heart Surgery nonclassifiable) were excluded from the cost-effectiveness and humanitarian footprint calculations. Every country where the charity teams were deployed to provide training and surgical patient care during 2015 was included.

### Cost Analysis

Service costs (logistical and clinical) sustained by the charity during the 2015 surgical campaign were included in the calculation of the overall costs per surgery. Values included in the computations were extracted from tax forms and financial reports and are expressed in 2015 US dollars.

All service expenses directly related to the intervention, including staff salaries, traveling and housing expenses, gifts in kind, volunteers’ time, donated equipment, medicines and disposables, costs of shipping the material from the central warehouse, reimbursements to staff and volunteers for excess luggage (hand-carried donations), and medical contracts (when staff or volunteers were not available) were included. Administration costs of running the charity (office space and administrative costs) were excluded.

Staff salaries, medicines, disposables, and capital costs borne by the LMIC where the interventions took place were also excluded from the cost-effectiveness calculations.

### Calculating Averted Disability-Adjusted Life-Years 

The assessment of averted disability-adjusted life-years (DALYs) was calculated for each patient individually. A step-by-step explanation of the equation used to that end, as well as a patient example, can be found in the eAppendix in the Supplement.

As defined by the World Health Organization, a DALY is “the sum of the years of life lost (YLL) due to premature mortality in the population and the years lost due to disability (YLD) for people living with the health condition or its consequences.”^8^ We used DALYs in our calculations of YLL for untreated patients and again as YLL after treatment owing to progression of the disease or as a sequel of surgical treatment. We used published data on age-weighted disabilities for the most common treated diagnoses—atrial septal defect, ventricular septal defect, d-transposition of the great arteries, tetralogy of Fallot, pulmonary stenosis, and cardiac diagnoses requiring complex palliative surgery (eg, single ventricle)—and for the congestive heart failure at the end of life that any untreated patients would have had if they remained untreated.^9^ These concepts (DALY, YLL, and YLD) are relatively new and therefore absent from the historical pediatric cardiology reference textbooks available. When disability weights were unavailable (eg, complex or uncommon diagnosis), a consensus was achieved by an ad hoc group (Table 1).

**Table 1.** Disability Weights After Surgical Treatment

Diagnosis	DALY Lost Due to Treatment
Atrial septal defect	0.03^a^
Ventricular septal defect	0.03^a^
Tetralogy of Fallot (treated during childhood or adolescence)	0.2^a^
Transposition of the great arteries (treated during childhood or adolescence)	0.2^a^
Tetralogy of Fallot (treated as young adult)	0.11^a^
Transposition of the great arteries (treated as young adult)	0.11^a^
Pulmonary stenosis (treated during childhood or adolescence)	0.02^a^
Pulmonary stenosis (treated as young adult)	0.16^a^
Palliated complex congenital heart disease (eg, palliative surgery for diagnosed single ventricle—all variants)	0.72^a^
Complete atrioventricular canal	0.1^b^
Subaortic stenosis	0.05^b^
Truncus arteriosus or aortopulmonary window	0.2^b^
Patent ductus arteriosus	0.01^b^
Ebstein malformation	0.5^b^
Supravalvar mitral stenosis	0.02^b^
Aortic arch hypoplasia with ventricular septal defect	0.5^b^
Supravalvar aortic stenosis	0.01^b^
Coarctation of the aorta	0.01^b^
Total anomalous pulmonary venous connection	0.03^b^
Mitral valvuloplasty	0.2^b^

Abbreviation: DALY, disability-adjusted life-year.

^a^ Published DALYs lost owing to surgical treatment.^9^

^b^ Consensus DALYs lost owing to surgical treatment.

Once DALYs averted for each patient according to his or her specific diagnosis and country of origin were accurately accounted for, estimating potential extra years of LE with the consequent extra years of schooling and potential extra lifetime income earnings was a relatively straightforward process using the following formula: quality-adjusted life-year = 1 − DALY. The resulting DALYs averted were not discounted at the standard 3% rate, and the implications of this choice are discussed later in the article.

### Calculating Years of Schooling and Future Income

Calculations for potential extra years of schooling assumed 6 years as minimum age to begin school regardless of country of birth. Calculations for potential extra income resulted from multiplying the published average yearly gross national income per capita in the country where the intervention took place by the extra years of LE gained by each survivor. Age weighting was not used in the calculations of potential future incomes. The present value of future dollars was calculated using a 3% discounting rate.

### Cost-effectiveness Analysis

Once DALYs averted for the entire cohort were known, the DALYs lost due to the negative effects of surgery were subtracted. The DALYs averted were averaged among all patients by dividing the total DALYs by the number of patients. Knowing the cost of a surgery (in 2015 US dollars) and the average DALYs averted per patient, we calculated the cost-effectiveness of the intervention per each DALY averted.

### Humanitarian Footprint of the Intervention

#### HDI Indicators

According to the United Nations definition, the HDI “is a summary measure of average achievement in key dimensions of human development.”^5^ These dimensions include population mean life expectancy as a proxy for a country’s health, median years of schooling as a measure of a population’s ability to acquire knowledge, and the gross national income per capita as a reflection of a country’s standard of living. Estimates were based on the preliminary country HDI data for 2014, which were downloaded in August 2016. Since then, an updated profile for each country has been published on the same United Nations website (Table 2).

**Table 2.** Human Development Indicators, Used to Calculate the Humanitarian Footprint

Country	LE-1, y	LE-2, y	MYS-1, y	MYS-2, y	GNIpc-1, $	GNIpc-2, $	HDI Rank-1	HDI Rank-2
China	75.3	75.8	7.5	7.5	11 477	12 547	91	90
Macedonia	75.2	75.4	8.2	9.3	11 745	11 780	84	81
Honduras	73.8	73.1	5.5	5.5	4138	3938	129	131
Iran	74.0	75.4	7.8	8.2	13 451	15 440	75	69
Iraq	69.4	69.4	5.6	6.4	14 007	14 003	120	121
Libya	75.3	71.6	7.5	7.3	21 666	14 911	55	94
Nigeria	52.5	52.8	5.2	5.9	5353	5341	152	152
Pakistan	66.6	66.2	5.2	4.7	4652	4866	146	147
Russia	68.0	70.1	11.7	12.0	22 617	22 352	57	50
Ukraine	68.5	71.0	11.3	11.3	8215	8178	83	81

Abbreviations: GNIpc-1, gross national income per capita at the time data were analyzed; GNIpc-2, updated GNIpc values; HDI rank-1, country ranking on the Human Development Indicators at the time of original data captured; HDI rank-2, updated HDI rankings; LE-1, life expectancy at the time data were analyzed; LE-2, updated LE values; MYS-1, median years of schooling at the time data were analyzed; MYS-2, updated MYS values.

#### Calculating the Patients’ Natural History According to Their Diagnosis

Projections of the natural history for each patient, if left untreated, are challenging for Western-trained clinicians who are no longer exposed to patients having late diagnosis and treatment. Every effort was made to find published data on the natural history of each particular congenital heart diagnosis as cited in historical pediatric cardiology reference textbooks and specialty journals.^10,11,12,13,14,15,16,17,18,19,20,21,22,23,24,25,26,27^

For complex cardiac malformations that were rarely diagnosed at the time historical reference textbooks were printed, an ad hoc group of 4 clinicians, 2 senior surgeons, and 2 senior cardiologists reviewed each diagnosis independently. Based on personal experience and historical frame of thought, each physician decided on a generic, albeit subjective, maximum LE one would anticipate for each of those patients if untreated. Despite the subjectivity of the methodology, most of these patients were assigned similar hypothetical LEs by all 4 members of the group. When variations in criteria were present, they never exceeded a 5-year time frame. Discrepancies were solved by calculating the median between the most extreme values. A list of common diagnoses with their published natural history as well as the consensus expected natural history for less common or complex congenital heart defects is presented in Table 3. The same ad hoc group contributed the estimated DALYs lost after treatment when published data were not available.

**Table 3.** Estimated Values Used to Calculate the Natural History of Patients (Life Expectancy) if Surgical Treatment Was Not Available

Diagnosis	Natural History if Untreated
Atrial septal defect	50 y
Partial anomalous pulmonary venous connection	50 y
Total anomalous pulmonary venous connection	
Infracardiac	1 y
Cardiac and supracardiac	50 y
Atrioventricular canal	
Complete	2 y
Partial	50 y
Transitional	50 y
Ventricular septal defect	50 y
Patent ductus arteriosus	50 y (median)
Tetralogy of Fallot	10 y (median)
With pulmonary atresia	10 y (median)
Severe pulmonary stenosis (isolated)	10 y
Pulmonary atresia and intact ventricular septum	6 mo
Tricuspid atresia	10 y
Ebstein anomaly of tricuspid valve	
Neonatal presentation	5 y
Adolescent presentation	25 y
Truncus arteriosus	1 y
Aortic stenosis	
Valvar	18 y (median)
Supravalvar	20 y
Subvalvar	30 y
Aortic insufficiency	15 y
Coarctation of the aorta	45 y
Coarctation and ventricular septal defect	1 y
Hypoplastic aortic arch and ventricular septal defect	1 y
Interrupted aortic arch	1 y
Transposition of the great arteries (simple)	1 mo
Transposition of the great arteries and ventricular septal defect	1 y
Corrected transposition of the great arteries	20 y (median)
Double outlet right ventricle	50 y
Double outlet right ventricle and pulmonary stenosis	10 y
Single ventricle (nontricuspid atresia)	4 y
Mitral stenosis	
Supravalvar mitral ring	6 y
Parachute mitral valve	10 y
Typical congenital mitral stenosis	6 mo
Mitral insufficiency	10 y
Hypertrophic cardiomyopathy	50 y

## Results

During 2015, a total of 446 patients underwent surgical treatment during 26 consecutive or concomitant 2-week trips to centers in 10 LMICs. The cohort included 192 female patients (43%) and 254 male patients (57%) with a mean (SD) age of 3.7 (5.4) years and 156 patients younger than 1 year, including 37 newborns. The mean (SD) weight of the patients in the cohort was 13.3 (12.1) kg, with 23% of patients weighing less than 5 kg. The overall program service costs for 2015 were $3 210 873, and the estimated cost per surgery was $6831.

Twenty-two patients underwent an unplanned reoperation and 2 patients underwent 2 reoperations during the same hospitalization, with an acceptable reoperation rate of 5.3%.^28^ The costs for the patients who underwent reoperation increased to $13 662 (n = 22) and $20 493 (n = 2).

Of the 446 patients, 16 were adults with CHD diagnoses and 6 pediatric patients had surgical procedures for diagnoses not included in the Risk Adjustment for Congenital Heart Surgery classification. These 22 patients, while included in the overall surgical costs calculations, were excluded from the cost-effectiveness analysis and the calculations of the humanitarian footprint, bringing the size of the analyzed cohort to 424 children younger than 16 years undergoing surgical treatment for their heart condition.

The overall unadjusted mortality rate was 8% (34 of 424). Our calculations took into consideration the negative effects of surgery on the LE calculations for the group, as some patients were expected to survive for at least a few more years even without surgery (n = 27), while excluding those with no chance of survival without a surgical procedure (n = 7). The negative effect of surgery on LE was 326 DALYs, bringing down the total number of DALYs averted to 39.9 per survivor. Given a cost per surgery of $6831, the cost-effectiveness of the intervention was $171 per DALY averted.

For pediatric patients who underwent reoperations during the same hospitalization, the cost-effectiveness was $342 per DALY averted. For the 2 patients who underwent 2 reoperations, it was $513 per DALY averted.

Regarding our secondary goal and after subtracting the negative effects of surgery for all 3 indicators, each patient in our cohort (390 of 424) potentially gained 39.9 years of LE, 3.5 years of schooling, and $159 533 gross national income per capita at purchasing power parity and at 3% discounting. This was equivalent to a humanitarian footprint of 16 932 years of LE added, 1484 years of schooling, and $ 67 642 191 gained by the entire cohort.

The largest program service expenses were the estimated wages lost by the 378 volunteers (a total of $1 479 014 assuming that all volunteers would be paid according to US Department of Labor tables)^29^ and donated medicines and disposables ($443 652). These 2 cashless transactions represented nearly 60% of the all expenses. Other significant service costs included team travel and boarding ($419 348), contracted medical services ($320 094), and clinical staff (n = 12) salaries and benefits ($364 541). The remaining costs ($184 224) included smaller amounts spent in 1-time contracts, shipping costs, other medical expenses, and bank fees.

There were 27 uniquely identifiable cardiac malformations, with the 5 most common being atrial septal defect, ventricular septal defect, patent ductus arteriosus, tetralogy of Fallot, and single ventricle. The number of surgical procedures performed according to risk-adjusted categories is listed in Table 4.

**Table 4.** Number of Patients Younger Than 16 Years Operated on by Risk-Adjusted Congenital Heart Surgery Categories

Risk-Adjusted Congenital Heart Surgery Category^a^	Patients, No.	Mortality, No. (%)
1	80	0
2	171	11 (6.4)
3	134	17 (12.6)
4	39	6 (10.2)
Total	424	34 (8.0)

^a^ The Risk Adjustment in Congenital Heart Surgery method is a systematic categorization of the complexity of the cases in which 1 corresponds to the lowest complexity and 6 to the highest.

## Discussion

Over the last 20 years, child mortality around the world has decreased significantly,^30^ yet with nearly 1.3 million children born with CHD every year in areas with inadequate or absent services,^31^ disability and early deaths have, in relative terms, consistently increased. Congenital heart disease is among the top 10 causes of YLL in Latin America, Central Asia, Africa, and the Middle East, with nearly 250 000 neonates and infants lost every year owing to lack of early diagnosis and/or treatment.^32^ Birth defects in general, among which CHD accounts for at least 50%, will continue to be among the top 15 causes of the global burden of disease for the first quarter of the current century,^33^ and their management in underserved areas of the world is limited to the sparse efforts of local governments and homegrown or international humanitarian organizations.

To our knowledge, the cost-effectiveness of pediatric cardiac surgery in LMICs has not been studied in detail before, but there is limited information available on the costs. Described within a range of $3000 to $10 000 per surgery,^34^ these costs are closer to the findings of this study and far from the common surgical charges at Western academic centers.^35^

A recent systematic review of the cost-effectiveness of essential surgical services in LMICs by Grimes et al^36^ found a number of global basic surgical interventions (cataract surgery, male circumcision, emergency cesarean delivery, and cleft palate surgery) to be as cost-effective as oral rehydration, promotion of breastfeeding, and antiretroviral therapy for HIV.

These findings are corroborated by a contemporary review by Chao et al^37^ with cost-effectiveness values as low as $13.78 per DALY for circumcision, $108.74 per DALY for hydrocephalus, $136 per DALY for ophthalmic surgery, and $82.32 per DALY for other common general surgical procedures.

These views are further reinforced by Saxton et al,^38^ who went so far as to include and define congenital heart surgery in LMICs as an “essential pediatric surgical procedure” because it offers considerable economic value. In fact, pediatric cardiac surgery seems to be at the same cost-effectiveness level as many currently funded health interventions,^39^ even when reoperations are required.

Cost-effectiveness analysis may be a perfectly appropriate tool to be used in the context of prevention of communicable diseases, for which it was originally designed, but could be considered inadequate for the assessment of surgical interventions, where prevention, incidence, and prevalence do not follow the patterns of communicable diseases.^40^ Conceivably, as countries move through their epidemiological transition, we should consider the development of new tools, different than the ones used when communicable diseases led among the causes of death. One potential approach would be to apply the long-term effect concept of “footprint of human action,” originally introduced in 1992 in the ecological literature^41^ (eg, carbon footprint), which we adapted to the humanitarian footprint concept.

The humanitarian footprint may be better at expressing the remarkable developmental impact that timely global health interventions in general, and surgical humanitarian efforts in particular, may have in LMICs, as it can better account for the long-term societal gains in human development. Thus, we advocate the use of alternative tools like this one to study global humanitarian health interventions for noncommunicable diseases, as it may lead to improved allocation of our limited global resources.

### Limitations

#### Program Sustainability

Dwelling on the logistic details of global humanitarian interventions is not the goal of a cost-effectiveness analysis, yet the most cost-effective interventions would not serve their purpose if the programs implemented were not sustainable over time.

Historically, the global charity involved in this study has initiated program development initiatives only as a result of a specific invitation by a local ministry of health, a local medical center, or a local charity. The vetting process implemented before the program planning phase has 2 goals: local inspection of the clinical facilities where the intervention will take place and determination of availability of basic services for the population to be served. These include an urban setting, running water and electricity at home, availability of some form of accessible transportation for follow-up clinic visits, and the provision of medical services free of charge to the population served.

Most of these programs are set within a country’s national health system facilities and provide free care to children, with the only potential affordability limitation being whether the family may need special transportation funds to reach the clinical facility for long-term follow-up. The sustainability of programs implemented by the charity since 1994 has been documented in specialty journals.^42,43,44^

#### Diagnosis Variability

With over 150 potential uniquely distinguishable anatomic diagnoses and more than 100 distinctive surgical procedures, our results could be criticized for treating CHD as a single disease and its treatment as a single treatment. Accordingly, assigning DALYs in concordance to individual diagnoses might have proven to be more accurate than averaging DALYs averted by all patients. Indeed, before we averaged the results, our calculations gave variable results for different diagnoses. For instance, if we were to compare 2 patients from the same country, 1 born with a ventricular septal defect operated on at the age of 3 years and 1 born with a d-transposition of the great arteries but operated on at the age of 6 days, the discrepancy in gains would be significant at the individual level. The patient with a ventricular septal defect (who could reach early adulthood, albeit with disabilities) would have significantly lower gains in human development indicators than the patient with transposition of the great arteries (a malformation with an LE of a few days if untreated).

As a matter of public health equity, there should be a sense of urgency to treat CHD as a single diagnosis. Furthermore, while diagnosis may vary at the individual patient level, the resources needed to treat children born with CHD do not. A proper hospital setting, qualified human resources, and adequate funding are needed regardless of the type of congenital heart defect. Every child born with CHD should have an opportunity for treatment, and the decision as to who receives treatment should not be an economic or a political one, but rather a medical and ethical one made by trained specialists.

#### Budgeting the Costs of Reoperations

Unplanned reoperations are an unwanted yet permanent feature of this surgical specialty, and budgeting allowance for those cases requiring more than 1 intervention should be considered during the planning phase of the health intervention. While our results discriminate cost-effectiveness according to the number of reoperations, in real-life situations, it would be safer to assume that a small percentage of patients will undergo reoperations during the same hospitalization and to budget accordingly.

#### Negative Effects of Surgery

Surgical mortalities from a purely economics perspective could be divided into 2 categories: (1) patients who were not expected to survive without surgical treatment and (2) patients who even without surgery would have survived a few years. The former category does not affect long-term estimates of the humanitarian footprint (surgery was the only hope for survival for these patients), but the latter category would have a negative impact on the calculations.

Twenty-seven mortalities fell into the second category, with a cumulative negative effect of 326 years of LE not realized, 184 years of schooling lost, and $3 018 411 in potential lifetime earnings lost because of the unsuccessful result of the surgery. These negative effects were subtracted from the positive effects of surgery on the survivors and are reflected in the results.

#### Discounting and Age Weighting Regarding DALYs Averted

The use of discounting and age weighting when calculating DALYs averted is the recommended standard for cost-effectiveness analysis according to the World Health Organization.^45^ While discounting at a predetermined rate is standard practice in economic studies when calculating costs and benefits, it has not been easily embraced in the health care sector for several reasons, ethical issues being one of them.^46^ The use of DALYs in general has been criticized for disfavoring children and measuring societal benefits over an individual’s life,^47^ and the use of discounting is controversial because “health, unlike wealth, cannot be invested to produce future gains.”^48^

Regardless of the reservations we might have about using discounted averted DALYs in the cost-effectiveness calculations for this study, we must be cognizant that not discounting DALYs may have led to an underestimation of incremental cost-effectiveness ratios (ie, better results).^49^ Even after halving the DALYs averted, which would happen if age weighting and discounting are used in the averted DALYs calculations, the results would continue to be highly cost-effective for the countries where the interventions took place.

#### Costs Excluded

Local personnel, infrastructure, and locally obtained medications and disposables were not included in this study. A critical view may assess that had these costs been included, our cost per surgery would have been higher, reducing the cost-effectiveness of the intervention. At the sites where the interventions took place, a limited number of local staff were actually hired (more commonly, staff were reassigned while keeping similar status on the payroll), and no significant infrastructure was actually built because of a specific request by the charity. To have a realistic perspective on the influence of local costs on the intervention, the salary of a cardiac surgeon in the countries included, with the exception of Iran, ranges from $200 to $1500 per month in 2015 US dollars. Nurses’ salaries are even lower, ranging from $100 to $500 per month. Local disposable material and medication, while not negligible, add little cost to the intervention because most of these materials are carried in or shipped in advance by the charity, with the exception of intravenous fluids and a few minor intensive care unit supplies (eg, generic antibiotics). The only country where all supplies were provided locally, except for certain specialty medications brought in by the charity, was Iran.

An intervention in LMICs is considered cost-effective as long as the cost per DALY averted is lower than the annual gross national income.^50^ The overall costs of this intervention would need to increase significantly to surpass $4000 per DALY averted (the lowest annual income per capita of the countries where operations took place in 2015) and to become not cost-effective.

The true impact of our interventions may still be unknown despite our best efforts, as there are other direct and indirect costs and/or benefits not assessed. For instance, the cost of routine cardiology follow-up of these patients after surgery for the rest of their lives with the consequent expenses for a family of limited resources, the cost savings of a patient who without treatment would have required frequent hospitalization in an already strained system, or the potential changes to the fertility rate due to increased survivorship of children beyond the first year of life were not accounted for. These and many other variables were not integrated into our analysis because of the difficulty of establishing an accurate estimate of these costs and benefits.

## Conclusions

Humanitarian global interventions aimed at the establishment of pediatric cardiac surgery programs in LMICs are highly cost-effective and deserving of stronger international support. While cost-effectiveness constitutes a good metric for humanitarian interventions regarding contagious diseases, perhaps more encompassing measures, like our humanitarian footprint, should be considered when evaluating global surgical interventions.

## Notes

Supplement. **eAppendix.** Equation Used in Calculating DALYs Averted and Patient Example
