# Supplementary material for: Cost-effectiveness of Humanitarian Pediatric Cardiac Surgery Programs in Low- and Middle-Income Countries
Source: JAMA Netw Open. 2018 Nov 16;1(7):e184707. doi: 10.1001/jamanetworkopen.2018.4707 (PMC6324367; doi:10.1001/jamanetworkopen.2018.4707)
Supplement: Supplement. — eAppendix. Equation Used in Calculating DALYs Averted and Patient Example [file jamanetwopen-1-e184707-s001.pdf]

Supplementary Online Content

Cardarelli M, Vaikunth S, Mills K, et al. Cost-effectiveness of humanitarian pediatric cardiac surgery programs in low- and middle-income countries. *JAMA Netw Open*. 2018;1(7):e184707. doi:10.1001/jamanetworkopen.2018.4707

eAppendix. Equation Used in Calculating DALYs Averted and Patient Example

This supplementary material has been provided by the authors to give readers additional information about their work.

© 2018 Cardarelli M et al. *JAMA Network Open.*

eAppendix. Equation Used in Calculating DALYs Averted and Patient Example

<u>Equation used in calculating DALYs averted</u>

(f – {[a – (a x **b)] – d}) - {(f – {[a – (a** x **b)] – d}) - (f – {[a – (a** x **b)] – d})** x **h} = i**

(f – {c – d}) – **{** (f - { c– d}) - (f – { c – d}) x h **}** = i

(f – e) – { (f – e) - (f – e) x h } = i

g – { g – (g x h) } = i

<u>Where:</u>

a = Natural history of a specific diagnoses

b = DALYs for end stage heart failure for untreated CHD (0.35 of the natural history)

a – (a x b) = c (True Life Expectancy for that specific diagnosis w/o treatment)

<u>and where:</u>

d = Age at time of surgery

c – d = e (Life Expectancy for a specific patient with a specific diagnosis W/O treatment)

<u>with:</u>

f **=** Median Life Expectancy for country of origin for that particular patient

f – e = g (years of disability or early death avoided by surgery)

<u>and where:</u>

h = DALYs lost even after treatment, due to need for future re-operations, complications of the surgery, progression of the disease, etc., presented as a percentage of time)

g – (g x h)= i (actual number of DALY averted for each specific patient according to the country of birth and adjusted for patient’s age at time of treatment)

© 2018 Cardarelli M et al. *JAMA Network Open.*

Patient example

Child born in Iraq diagnosed with Transposition of the Great Arteries with Ventricular Septal Defect and operated on day 14 of life.

a = 1 year (from Table 3)

b = 0.35

a – (a x b) = c 1 year – (1 year x 0.35) = 1 year – 0.35 = **0.65 year**

and where:

d = 14 days

c – d = e = 0.65 year – 14 days = 237 days – 14 days = 223 days or **0.61 year**

with:

f **=** 69.4 years (from Table 2)

f – e = g 69.4 – 0.61 = **68.7 years**

and where:

h = 0.20 (from Table 1)

g – (g x h)= i = 68.7 – (68.7 x 0.20) = 68.7 – 13.74 = **54.96** DALYs averted for this patient

© 2018 Cardarelli M et al. *JAMA Network Open.*
